# Evaluation of an educational intervention in oral health for primary care physicians: a cluster randomized controlled study

**DOI:** 10.1186/s12903-018-0676-2

**Published:** 2018-12-14

**Authors:** Simin Z. Mohebbi, Sepideh Rabiei, Reza Yazdani, Pentti Nieminen, Jorma I. Virtanen

**Affiliations:** 1 Research Center for Caries Prevention, Dentistry Research Institute, Tehran University of Medical Sciences, Tehran, Iran; 2 Department of Community Oral Health, School of Dentistry, Tehran University of Medical Sciences, P.O. Box 1439955991, Tehran, Iran; 3 Medical Informatics and Statistics Research Group, University of Oulu, P.O. Box 5000, FI-90014 Oulu, Finland; 4 Department of Clinical Dentistry, Faculty of Medicine, University of Bergen, P.O. Box 7804, N-5020 Bergen, Norway; 5 Medical Research Center, Oulu University Hospital, P.O. Box 21, FI-90029 Oulu, Finland

**Keywords:** Educational, Intervention, Oral health, Primary care, Physicians

## Abstract

**Background:**

Family physicians are in frequent contact with patients, and their contribution to oral health promotion programs could be utilized more effectively. We implemented an oral health care (OHC) educational seminar for physicians and evaluated its impact on their knowledge retention in OHC.

**Methods:**

We conducted an educational trial for primary care physicians (*n* = 106) working in Public Health Centers in Tehran city. W*e launched a* self-administered questionnaire about pediatric dentistry, general dental, and dentistry-related medical knowledge and backgrounds. Physicians in intervention group A (*n* = 38) received an educational intervention (Booklet, Continuous Medical Education (CME), and Pamphlet), and those in group B (*n* = 32) received only an OHC pamphlet. Group C (*n* = 36) served as the control. A post-intervention survey followed four months later to measure the difference in the physicians’ knowledge; the Chi-square test, ANOVA and linear regression analysis served for statistical analysis.

**Results:**

The intervention significantly increased the physicians’ oral health knowledge scores in all three domains and their total knowledge score (*p* <  0.001). Those physicians who had lower knowledge scores at the baseline showed a higher increase in their post-intervention knowledge. The models showed no associations between the background variables and the knowledge change.

**Conclusion:**

The primary care physicians’ OHC knowledge improved considerably after an educational seminar with a reminder. These findings suggest that OHC topics should be included in physicians’ CME programs or in their curriculum to promote oral health, especially among non-privileged populations.

## Background

Oral disease may manifest in a variety of forms including including dental caries, periodontal disease and oral mucosal lesions including oral cancer [1]. They pose a serious problem in communities and negatively impact individual quality of life, particularly in low-income populations [1, 2]. Since primary care providers and family physicians are in frequent contact with patients, engaging them would be an efficient approach in oral health promotion programs [2]. In addition, the common risk factor approach (CRFA) considers common health determinants for preventive measures in a multi-professional collaboration [3]. A number of recent reports have emphasized the importance of integrating the dental and medical professions and involving all health professionals in public oral health care (OHC) [4–8]. Although physicians have shown a willingness to learn about OHC and to provide preventive measures for their patients [5, 9], they receive limited training in this field, and medical curricula often include only limited information on OHC [5, 7, 10–13].

Previous studies have examined the evidence related to oral health promotion and showed oral health education programs to improve primary care staff’s knowledge of oral health issues [14–17]. Moreover, research has shown that specifically planned oral health care education increases physicians’ knowledge and may help them promote children’s oral health among deprived populations in developed countries [7, 15]. Knowledge retention after educational interventions, however, remains uncertain [16].

Research has shown continuous medical education (CME) to be an effective way to increase physicians’ knowledge and to improve the provision rate of preventive dental services by pediatric health care providers. The effectiveness of such programs in countries with developing health care systems and where primary care physicians in the position of family physicians for deprived populations has not yet been studied [10, 13, 17, 18].

## Methods

Our aim was to implement an evidence-based OHC interactive educational seminar for physicians and analyze its impact on the physicians’ retention of OHC knowledge.

### Study design and subjects

Tehran has seven District Health Centers (DHC), three of which are located in the non-affluent area where this study was carried out. Each DHC supervises 15 to 30 public health centers with varying numbers of physicians [1–4] in each center. The three DHCs in the non-affluent area were randomly assigned to two intervention (A and B) groups and one control (C) group. All physicians in each DHC were invited to take part in the study. We *sent to the physicians a* self-administered questionnaire enquiring about dental knowledge through the administrative DHC along with a gift of toothbrush and dental floss. The physicians had to return the questionnaires in 48 h. Two reminders were sent to those who had not returned their questionnaire to their DHC in time. In all, 106 physicians participated in both baseline and post-intervention studies. The number of physicians in group A was 38, in group B, 32 and in group C, 36*.* We collected the baseline data from April to May 2011 and the outcome data 4 months later.

### Educational interventions

For the intervention, we created an evidence-based booklet [18–21] consisting of six parts: oral health and diseases in adults, oral health and diseases in children, oral health in pregnancy, fluoride and dental caries, dental emergency, the relationship between oral and systemic diseases. We also prepared a pamphlet that included the most important parts of the booklet; both were in Persian.

The educational interventions were as follows:

- **Group A** (Booklet, Continuous Medical Education (CME), and Pamphlet): The physicians received the booklet after the baseline survey. The CME seminar was arranged 2 weeks later by their DHC. The half-day seminar by one of the authors (S.R.) provided the physicians with a lecture (power point slides) on the topics of the booklet and pamphlet, blended with case-based education, followed by a discussion of preventive approaches followed by a question and answer session. Another CME seminar was held in the same place 1 week later for those physicians who could not take part in the first one. After one and a half months and with the help of the DHC health education deputies, we distributed the pamphlet to the intervention centers as a reminder.
- **Group B** (Pamphlet Only): Physicians in this group received only the pamphlets distributed to the centers by the DHC staff. This group received neither the educational seminar nor the booklets.
- **Group C** (Control): The control group received no OHC information during this four-month period. After the trial, these physicians also received the booklet and the pamphlet.

### Questionnaire

The structured questionnaire of the study was based on previously validated surveys [9, 13, 17] with minor modifications [5]. The questionnaire evaluated knowledge in three domains: a) pediatric dentistry knowledge, b) general dental knowledge, and c) dentistry-related medical knowledge. Each domain included six questions (see Table 1). The responses were on a five-point Likert scale with alternatives ranging from “strongly agree” to “strongly disagree,” including “don’t know”. We dichotomized the answers to the questions to “1” for correct answers and to “0” for false and “don’t know” answers.

**Table 1:** Percentage (frequency) of physicians’ (*n* = 106) correct responses to knowledge questions

	Group A (*n* = 38)			Group B (*n* = 32)			Group C (*n* = 36)		
	Pre	Post	Diff	Pre	Post	Diff	Pre	Post	Diff
Paediatric domain									
The bacteria that causes dental decay usually transmit from mother to the child	16 (6)	84 (32)	68 (26)	22 (7)	31 (10)	9 (3)	33 (12)	42 (15)	33 (12)
Toothpastes which contain fluoride should not be used for children under 3 years old	18 (7)	53 (20)	35 (13)	22 (7)	13 (4)	-9 (−3)	17 (6)	17 (6)	17 (6)
Teeth cleaning and brushing should be started from 2- to 3- years old, when deciduous dentition is completed	21 (8)	55 (21)	34 (13)	38 (12)	47 (15)	9 (3)	36 (13)	25 (9)	36 (13)
Pacifier sucking in under-4-year-old children is a risk factor for dento-alveolar malformation	3 (1)	42 (16)	39 (18)	6 (2)	9 (3)	3 (1)	14 (5)	17 (6)	14 (5)
Using fluoride varnish on under-5-year-olds teeth causes fluorosis and poisoning	26 (10)	66 (25)	40 (15)	31 (10)	34 (11)	3 (1)	33 (12)	33 (12)	33 (12)
Sealants are effective in the prevention of pit and fissure caries in newly erupted molars	61 (23)	92 (35)	31 (12)	59 (19)	72 (23)	13 (4)	64 (23)	64 (23)	64 (23)
Dental domain									
Why fluoride is added to toothpaste	90 (34)	100 (38)	10 (4)	88 (28)	91 (29)	3 (1)	100 (36)	100 (36)	100 (36)
The first signs of decay are white spots or lines on teeth surfaces	47 (18)	97 (37)	50 (19)	47 (15)	63 (20)	16 (5)	44 (16)	39 (14)	44 (16)
The frequency of sugar consumption has a greater role in producing caries than does the total amount of sugar consumed	97 (37)	97 (37)	0 (0)	88 (28)	81 (26)	-7 (−2)	94 (34)	86 (31)	94 (34)
Using fluoride toothpaste is more important than the brushing technique for preventing caries	0 (0)	63 (24)	63 (24)	3 (1)	13 (4)	10 (3)	17 (6)	17 (6)	17 (6)
The best time to refer a pregnant woman for emergency dental procedure is in second semester	71 (27)	87 (33)	16 (6)	56 (18)	66 (21)	10 (3)	75 (27)	78 (28)	75 (27)
The main cause of periodontal diseases is dental plaque	82 (31)	100 (38)	18 (7)	72 (23)	84 (27)	12 (4)	86 (31)	89 (32)	86 (31)
Medical domain									
Periodontal diseases can cause LBW	61 (23)	90 (34)	29 (11)	75 (24)	78 (25)	3 (1)	64 (23)	75 (27)	64 (23)
Periodontal diseases can cause problems in diabetes control	63 (24)	97 (37)	34 (13)	75 (24)	75 (24)	0 (0)	75 (27)	78 (28)	75 (27)
Periodontal diseases can cause cardiovascular disease	79 (30)	90 (34)	11 (4)	94 (30)	92 (29)	−2 (−1)	92 (33)	92 (33)	92 (33)
Antidepressants increase the risk of dental caries	47 (18)	87 (33)	40 (15)	53 (17)	59 (19)	6 (2)	58 (21)	67 (24)	58 (21)
Analgesics increase the risk of dental caries	29 (11)	68 (26)	39 (15)	25 (8)	44 (14)	19 (6)	47 (17)	50 (18)	47 (17)
Antihypertensive drugs increase the risk of dental caries	13 (5)	71 (27)	58 (22)	28 (9)	34 (11)	6 (2)	28 (10)	31 (11)	28 (10)

Group A: Booklet + CME course + pamphlet, Group B: Pamphlet, Group C: Control, Pre: before, Post: after the intervention

Socio-demographics included the respondents’ age, gender, and working profile (public only / public and private practice). All the health centers were public, but some of the physicians also worked in the private sector after their health center shifts (public + private).

### Statistical analysis

We used IBM SPSS Statistics (IBM Corp. Released 2011. IBM SPSS Statistics for Windows, Version 20.0. Armonk, NY: IBM Corp.) for the statistical analyses. The chi-square test served to evaluate the statistical significance of differences in the background characteristics of the primary care physicians between the study groups. We used analysis of variance (ANOVA) to measure any improvement in knowledge as the main outcome between the intervention and control groups and linear regression analysis to estimate the independent effect of the interventions on the change in the physicians’ oral health knowledge scores after controlling for basic background characteristics.

### Ethics approval

Participation in the study was voluntary, and the responses were anonymous. All respondents provided their written informed consent. The Ethics Committee of Tehran University of Medical Sciences approved the study (The ethics approval reference number IR.TUMS.REC.1396.2949).

## Results

Altogether 121 physicians who were working in the public health centers of the non-affluent part of the city participated in the baseline study (response rate: 92%). Of these, 106 physicians completed and returned the outcome questionnaires (response rate: 88%) post-intervention (Group A: *n* = 38, Group B: *n* = 32, Group C: *n* = 36).

Women comprised a clear majority of the physicians in all three groups: 63% in group A, 78% in group B, and 75% in group C (*p* = 0.331). The mean age of the female physicians was lower than that of the males (35.4 years vs. 44.5 years; *p* <  0.001). Most of the women (80%) were working in the public sector only, whereas most of the men (70%) worked in both the private and public sectors (*p* <  0.001).

At baseline, only 3% of the physicians in group A knew the correct answer to the question “Pacifier sucking in under-four-year-old children is a risk factor for dento-alveolar malformation”; the percentage of correct answers for group B was 6%, and for the control group, 14%. Nobody knew that “Using fluoride toothpaste is more important than the brushing technique for preventing caries” in group A, while 3% in group B and 17% in control group answered correctly to this question.

Instead, 97% of physicians in group A, 88% in Group B, and 94% in control group knew that “The frequency of sugar consumption has a greater role in producing caries than does the total amount of sugar consumed” and 90% in group A, 88% in Group B, and 100% in control group knew “the reason of adding fluoride to toothpaste”. The percentage and frequency of the physicians’ correct responses to each knowledge questions in the three domains at the baseline and outcome data collection are shown in Table 1 for the two intervention and control groups.

In group A, the highest differences (68%) in the physicians’ correct responses before and after the intervention was related to the question “The bacteria that cause dental decay usually transmit from mother to the child” followed by; “Using fluoride toothpaste is more important than the brushing technique for preventing caries” with 63% difference; “antihypertensive drugs increase the risk of dental caries” with 58% difference; and “The first signs of decay are white spots or lines on teeth surfaces” with 50% difference.

The highest difference in percentage of correct answers was 19% in Group B and 11% in control group occurring for one single question in medical domain. The difference was “0” for 6 questions in control group.

The mean difference between knowledge total scores before and after the intervention was higher in Group A compared to the Control group in all three domains (*p* <  0.001) but not in any domain for group B compared to the control (Table 2).

**Table 2:** The changes in the knowledge scores among the physicians (*n* = 106) by the study groups

Domains	Change in scores before and after intervention			Significance	
	Group A (*n* = 38)	Group B *n* = 32	Control *n* = 36	*P*-value A vs C^a^	*P*-value B vs C^a^
	Mean (SD)	Mean (SD)	Mean (SD)		
Total Knowledge	6.16 (3.67)	1.03 (2.30)	0.19 (2.20)	< 0.001	0.375
Pediatric domain	2.47 (1.69)	0.28 (0.85)	0.00 (0.71)	< 0.001	0.524
Dental domain	1.58 (1.77)	0.44 (0.80)	− 0.08 (0.94)	< 0.001	0.062
Medical domain	2.11 (1.62)	0.31 (1.50)	0.28 (0.94)	< 0.001	0.992

Group A: Booklet + CME course + pamphlet;

Group B: Pamphlet;

Group C: Control;

^a^*P*- value of Dunnett’s multiple comparison test

Table 3 shows that when background factors and intervention grouping were included in the linear regression models predicting the outcome variables, group A intervention clearly increased the oral health knowledge scores in all three domains and the total knowledge score (*p* <  0.001). In addition, the physicians who had acquired lower knowledge scores at the baseline, revealed higher increase in their knowledge after the intervention. The models revealed no associations between the background variables (age, gender, working profile) and the knowledge change.

**Table 3:** Linear regression models for predicting change in the total knowledge score and the three domains

Explanatory variables	Standardized regression coefficient Beta	*P*-value of Wald test	95% confidence interval for Beta	
			Lower bound	Upper bound
Model 1: total score				
Group A intervention	0.64	< 0.001	0.377	0.655
Group B intervention	0.035	0.677	−1.14	1.75
Age	0.06	0.525	− 0.06	0.11
Gender^a^	0.09	0.311	− 0.77	2.39
Working place^b^	0.09	0.306	− 0.66	2.07
Total score at baseline	−0.30	< 0.001	− 0.63	− 0.20
Model 2: paediatric domain score				
Group A intervention	0.69	< 0.001	−1.62	2.74
Group B intervention	0.03	0.723	−0.49	0.70
Age	0.04	0.672	−0.03	0.04
Gender^a^	0.06	0.539	− 0.46	0. 88
Working place^b^	0.11	0.235	−0.23	0.91
Paediatric score at baseline	−0.17	0.0350	−0.43	−0.08
Model 3: dental domain score				
Group A intervention	0.58	< 0.001	−1.02	1.92
Group B intervention	0.19	0.235	−0.19	0.78
Age	0.09	0.338	−0.02	0.04
Gender^a^	0.02	0.809	− 0.45	0.58
Working place^b^	0.04	0.684	−0.36	0.54
Dental score at baseline	−0.44	< 0.001	−0.74	− 0.34
Model 4: medical domain score				
Group A intervention	0.42	< 0.001	−0.82	2.14
Group B intervention	−0.04	0.696	−0.81	0.54
Age	0.04	0.657	−0.03	0.05
Gender^a^	0.11	0.262	− 0.32	1.15
Working place^b^	0.09	0.343	−0.33	0.94
Medical score at baseline	−0.42	< 0.001	−0.67	− 0.29

^a^Gender:female = 1, male = 2, ^b^Working place: public = 1, public+private = 2

## Discussion

Family physicians may play a crucial role in reducing oral health problems for their covered population. This randomized interventional study showed that an OHC educational seminar for the physicians effectively improved their OHC knowledge and their knowledge retention after 4 months. Integrating oral health promotion into existing preventive programs improves individual access to OHC prevention, especially among underprivileged groups [22].

Previous studies have shown that the oral health knowledge of physicians in various countries such as Canada, Iran, Italy, Saudi Arabia, and the USA is insufficient [5, 9, 10, 12, 13]. Historically, oral health has operated independently from medicine, and physicians seldom collaborate with dentists [23]. In most countries, children see a primary care physician about ten times for health screenings before their first birthday [24]. In addition, many at-risk groups do not regularly visit the dentist, whereas contact with medical practitioners, including family physicians, is more common [25].

Deepening physicians’ understanding of the relationship between the oral cavity and the rest of the body [24] and training them to perform oral examinations, counsel patients, and refer them to dentists when needed, primary care physicians can help reduce oral health disparities [26]. This information should also become part of the university medical curriculum or continuing medical education (CME) for recertification activities.

Since medical curricula often lack sufficient oral health education [26, 27], CME may be an opportune point for inclusion of this topic. CME in oral health for physicians would increase the number of professionals who are prepared to help with prevention and early recognition of oral diseases, especially for at-risk groups and target populations such as children. Various programs have been introduced in countries such as the USA to provide oral health CME for pediatricians [15]. Physicians in public health centers typically have very busy workdays, which may have kept them from studying the pamphlet at all. Despite this, the CME seminar provided them an opportunity to focus on the educational material they received.

The present physicians receiving the CME seminar, along with the booklet and pamphlet reminder, demonstrated greater knowledge than did the group who received the pamphlet only or the control group. Our result is consistent with the results of prior studies, which have shown an increase/improvement in physicians’ OHC knowledge after 2 hours of didactic training [28]. Another similar study revealed that physicians provided preventive services for their patients after receiving 90 min of CME in OHC [15]. Our findings may be related to the effect of the interactive educational seminar, which gave the physicians the opportunity to ask and get answers from the lecturer as well as clear examples of and demonstrative slides. Previous research has also shown that motivation is an important factor in learning [29], and that the motivating and learning approach has proved useful in dental settings [30]. In CME, the traditional seminar is considered the most common method, though it may carry the limitations of an inactive audience. In comparison to the traditional lecture-based, the interactive methods such as workshops, online courses, hands-on practical training, are more effective in transferring information and changing physicians’ practice [31, 32]. In our study, we eliminated the weakness of traditional seminar by holding an interactive session and using an educational booklet as a learner-centered method of self-study. Use of reminder pamphlet in our interactive seminar group could have served as a call to action to motivate the physicians and support knowledge retention among the participants [33].

Our study included DHCs located in the non-affluent part of the city*.* People in the non-affluent parts of the city cannot afford private care; consequently, access to private practice is more limited [27]. Therefore, the number of public health centers and patient turn-over is higher in the non-affluent regions. Another study showed that physicians working in the non-affluent area, who may have been treating children from high-risk population, were more willing to receive OHC information [5]. There was no gender or age difference in the physicians’ total knowledge score increase which is the same as in the research findings from Italy in which the knowledge increase was the same for both sexes [13]. Our questionnaire was validated in previous studies [5]. Due to their busy program, the physicians were permitted to complete the questionnaires on their own time. As all the questionnaires were anonymous, the physicians had no obligation to find the correct responses in any other way than through their own knowledge, thus avoiding any influence on the findings. Physicians are busy in their daily work, and this study suggests taking advantage of distance education to provide easy access and the potential for interaction in future studies.

## Conclusions

The primary care physicians in our study attained a higher level of OHC knowledge after participating in an educational program. These findings suggest that physicians’ CME programs and curricula should include OHC topics if they aim to promote oral health, especially among non-privileged populations.

## References

[1] Peterson PE, Kwan S (2004). Evaluation of community based oral health promotion and oral disease prevention. WHO recommendations for improved evidence in public health practice. Community Dent Health.

[2] Inglehart MR, Filstrup SL, Wandera A, Inglehart MR, Bagramian RA (2002). Oral health and quality of life in children. Oral health- related quality of life.

[3] Sheiham A, Watt RG (2000). The common risk factor approach: a rational basis for promoting oral health. Community Dent Oral Epidemiol.

[4] Rabiei S, Mohebbi SZ, Yazdani R, Virtanen JI (2014). Primary care nurses' awareness and willingness towards children's oral health care. BMC Oral Health.

[5] Rabiei S, Mohebbi SZ, Patja K, Virtanen J (2012). Physicians’ knowledge of and adherence to improving oral health. BMC Public Health.

[6] Thema LK, Singh S. Integrated primary oral health services in South Africa: The role of the PHC nurse in providing oral health examination and education. Afr J Prm Health Care Fam Med. 2013;5 10.4102/phcfm.v5i1.413.

[7] Mouradian WE, Schaad DC, Kim S, Leggott PJ, Domoto PS, Maier R, Stevens NG, Koday M (2003). Addressing disparities in children's oral health: a dental-medical partnership to train family practice residents. J Dent Educ.

[8] Ford CR, Foley KT, Ritchie CS, Sheppard K, Sawyer P, Swanson M, Harada CN, Brown CJ (2013). Creation of an interprofessional clinical experience for healthcare professions trainees in a nursing home setting. Med Teach.

[9] Prakash P, Lawrence HP, Harvey B, Maclsaac WJ, Limeback H, Leake JL (2006). Early childhood caries and infant oral health: pediatricians’ knowledge, practices and training. Paediatr Child Health.

[10] Lewis CW, Grossman DC, Domoto PK, Deyo RA (2000). The role of the pediatrician in the oral health of children: a national survey. Pediatrics.

[11] Al-Hussyeen A, Al-Sadhan S, Al-Dhalaan R, Al-Ghanim B (2003). Pediatricians’ knowledge and practices towards children’s preventive oral health care in Saudi Arabia. Egyptian dental journal.

[12] Sabbagh HJ, El-Kateb M, Al Nowaiser A, Hanno AG, Alamoudi NH (2011). Assessment of pediatricians dental knowledge, attitude and behaviour in Jaddah. Saudi Arabia JClinPediatr Dent.

[13] Di Giuseppe G, Nobile CG, Marinelli A, Angelillo IF (2006). Knowledge, attitude and practices of pediatricians regarding the prevention of oral diseases in Italy. BMC Public Health.

[14] Le P, Dempster L, Limeback H, Locker D (2012). Improving residents’ oral health through staff education in nursing homes. Spec Care Dentist.

[15] Slade GD, Rozier RG, Zeldin LP, Margolis PA. Training pediatric health care providers in prevention of dental decay: results from a randomized controlled trial. BMC Health Services Research. 2007;7. 10.1186/1472-6963-7-176. 10.1186/1472-6963-7-176 PMC2190767 17980021

[16] Marinopoulos SS, Dorman T, Ratanawongsa N, Wilson LM, Ashar BH, Magaziner JL, Miller RG, Thomas PA, Prokopowicz GP, Qayyum R, Bass EB (2007). Effectiveness of Continuous Medical Education. Evid Rep Technol Assess (Full Rep).

[17] Dela Cruz GG, Rozier RG, Slade G (2004). Dental screening and referral of young children by pediatric primary care providers. Pediatrics.

[18] Andeasen JO, Andeasen FM, Andersson L (2007). Text book & color atlas of traumatic injuries to the teeth.

[19] Pinkham J, Casamassimo PS, DJ MCT, Fields HW, Nowak AJ (2005). Pediatric dentistry infancy through adolescence.

[20] Roberson TM, Heymann HO, Switt EJ (2006). Art & Science of operative dentistry.

[21] Newman MG, Takei HH, Klokkevold PR (2006). Carranza FA editor emeritus. Clinical periodontology.

[22] Petersen PE, Pine CM, Harris R (2007). Inequalities in oral health: the social context for oral health. Community Oral health.

[23] Mouradian WE, Reeves A, Kim S, Evans R, Schaad D, Marshall SG, Slayton R (2005). An oral health curriculum for medical students at the University of Washington. Academic Medicin.

[24] Chu M, Sweis LE, Guay AH, Manski RJ (2007). The dental care of U.S. children: access, use and referrals by nondentist providers, 2003. J Am Dent Assoc.

[25] Macpherson LM, McCann MF, Gibson J, Binnie VI, Stephen KW (2003). The role of primary healthcare professionals in oral cancer prevention and detection. Br Dent J.

[26] Ramirez JH, Arce R, Contreras A (2010). Why must physicians know about oral diseases?. Teach Learn Med.

[27] Cohen LA. Expanding the physician's role in addressing the oral health of adults. Am J Public Health. 2013;103(3):408–12. 10.2105/AJPH.2012.300990 PMC3673507 23327256

[28] Pierce KM, Rozier RG, Vann WF (2002). Accuracy of pediatric primary care providers’ screening and referral for early childhood caries. Pediatrics.

[29] Overton Dickinson A, Mason J (2005). Community oral health education. Concepts in dental public health.

[30] Wilson TG (1998). How patient compliance to suggested oral hygiene and maintenance affect periodontal therapy. Dent Clin N Am.

[31] Forsetlund L, Bjørndal A, Rashidian A, Jamtvedt G, O'Brien MA, Wolf FM, et al. Continuing education meetings and workshops: effects on professional practice and health care outcomes. Cochrane Database Syst Rev. 2009;2. 10.1002/14651858.CD003030.pub2. 10.1002/14651858.CD003030.pub2 PMC7138253 19370580

[32] Garattini L, Gritti S, De Compadri P, Casadei G (2010). Continuing medical education in six European countries: a comparative analysis. Health policy.

[33] Faghihi SA, Khankeh HR, Hosseini SJ, Soltani Arabshahi SK, Faghih Z, Parikh SV, Shirazi M (2016). Improving continuing medical education by enhancing interactivity: lessons from Iran. J Adv Med Educ Prof.

